# Multi-planar 2.5D U-Net for image quality enhancement of dental cone-beam CT

**DOI:** 10.1371/journal.pone.0285608

**Published:** 2023-05-11

**Authors:** Kanghyun Ryu, Chena Lee, Yoseob Han, Subeen Pang, Young Hyun Kim, Chanyeol Choi, Ikbeom Jang, Sang-Sun Han

**Affiliations:** 1 Artificial Intelligence and Robotics Institute, Korea Institute of Science and Technology, Seoul, South Korea; 2 Department of Oral and Maxillofacial Radiology, Yonsei University College of Dentistry, Seoul, South Korea; 3 College of Information Technology, Department of Electronic Engineering, IT Convergence Major, Soongsil University, Seoul, Korea; 4 Department of Radiology, Harvard Medical School, Boston, MA, United States of America; 5 Department of Mechanical Engineering, Massachusetts Institute of Technology, Cambridge, MA, United States of America; 6 Department of R&D Performance Evaluation, Korea Health Industry Development Institute (KHIDI), Cheongju, South Korea; 7 Department of Electrical Engineering and Computer Science, Massachusetts Institute of Technology, Cambridge, MA, United States of America; 8 Division of Computer Engineering, Hankuk University of Foreign Studies, Yongin, South Korea; 9 Department of Radiology, Massachusetts General Hospital, Harvard Medical School, Boston, MA, United States of America; Chung-Ang University Gwangmyeong Hospital, REPUBLIC OF KOREA

## Abstract

Cone-beam computed tomography (CBCT) can provide 3D images of a targeted area with the advantage of lower dosage than multidetector computed tomography (MDCT; also simply referred to as CT). However, in CBCT, due to the cone-shaped geometry of the X-ray source and the absence of post-patient collimation, the presence of more scattering rays deteriorates the image quality compared with MDCT. CBCT is commonly used in dental clinics, and image artifacts negatively affect the radiology workflow and diagnosis. Studies have attempted to eliminate image artifacts and improve image quality; however, a vast majority of that work sacrificed structural details of the image. The current study presents a novel approach to reduce image artifacts while preserving details and sharpness in the original CBCT image for precise diagnostic purposes. We used MDCT images as reference high-quality images. Pairs of CBCT and MDCT scans were collected retrospectively at a university hospital, followed by co-registration between the CBCT and MDCT images. A contextual loss-optimized multi-planar 2.5D U-Net was proposed. Images corrected using this model were evaluated quantitatively and qualitatively by dental clinicians. The quantitative metrics showed superior quality in output images compared to the original CBCT. In the qualitative evaluation, the generated images presented significantly higher scores for artifacts, noise, resolution, and overall image quality. This proposed novel approach for noise and artifact reduction with sharpness preservation in CBCT suggests the potential of this method for diagnostic imaging.

## Introduction

Cone-beam computed tomography (CBCT) is widely used in the dental field for purposes ranging from disease diagnosis to preoperative simulation and surgical guide construction [1, 2]. CBCT can provide three-dimensional (3D) images of the targeted area with the advantage of lower dosage than multidetector computed tomography (MDCT) [3, 4]. However, in CBCT, due to the cone-shaped geometry of the X-ray source and the absence of post-patient collimation, more scattering rays deteriorate the image quality than is the case with MDCT. The low radiation dose also causes under-sampling of the signal, which results in more noise and artifacts [4, 5].

Deep learning (DL) based approaches for CBCT image improvement have recently emerged as a possible viable solution in clinics. While prior studies have demonstrated the possibility of improving CBCT through the use of DL by reducing artifacts and noise and by standardizing pixel intensity [6–12], DL inference is still limited by obscured anatomic fine details and blurred edges in the image [7, 13]. These factors may limit the diagnostic utility of CBCT for intricate features such as the teeth, alveolar bone pattern, sinuses, or the temporomandibular joint (TMJ) complex. Thus, developing a network that minimizes artifacts and noise while maintaining fine anatomic details is necessary for potential diagnostic usage.

Automated methods for 3D image synthesis, quality enhancement, and segmentation tasks are becoming increasingly important in the biomedical field. Various recent network architectures which demonstrated good performance in tooth segmentation (e.g., 3.5D U-Net) could also be explored for cone-beam artifact correction [14]. A common approach is to expand the U-Net structure to work with volumetric data by utilizing 3D convolutions [15]. However, this approach requires a huge memory and long training time. Additionally, medical images are often anisotropic and inconsistent in volume resolution across participants. This problem can sometimes be resolved by resampling the volumes to be isotropic [16, 17]. However, these methods only interpolate the data and often result in blurry images. DL methods therefore are often applied to 2D slice images, but these slice images do not contain information on the full 3D data, which makes the tasks challenging. One solution for incorporating information from the 3D surroundings is to train the model on orthogonal patches extracted from axial, sagittal and coronal views [18–20]; this approach is referred to as “multi-planar” in this paper. For instance, a multi-planar U-Net would predict a value for an intersecting voxel from three orthogonal slice images. Another remedy is to expand a 2D U-Net model to accept 3-channel data and supply the model with a slice and its adjacent slices (e.g., slices #N-1, #N, and #N+1 instead of slice #N) [7, 14]; the technique is referred to as “2.5D” in this paper. This method is particularly useful in our study when the input and output images are slightly misaligned.

Previous DL-based studies by Yuan et al. [7] and Chen et al. [8] presented a two-dimensional U-Net with L1 loss and focused on correcting the pixel values as Hounsfield units (HUs) for head and neck tumor localization to plan radiation therapy [6–8]. However, training a U-Net with a conventional loss function has limitations in that it computes pixel-wise error while assuming a precise alignment between the input (CBCT) and output images (MDCT). The problem is that their perfect alignment is quite difficult in the real world because MDCT scans are usually performed lying down, while CBCT scans are performed sitting or standing, which leads to differences in topology and the relative positions of the maxillary-mandibular and facial soft tissue between the two images. A slight misalignment can easily propagate to exacerbate blurring. In particular, pixel-wise L1 loss is known to be insufficient for considering critical local features such as minute details, resulting in blurry images [21].

We introduced and applied several advanced techniques to resolve the aforementioned issues. First, we built an automated data preparation process involving intensive image registration and erroneous background masking, which aimed to minimize errors in image registration. Second, we leveraged the two recent techniques introduced above—namely, the multi-planar and 2.5D methods—to efficiently train the model with limited hardware resources while allowing slight misalignments between the images. Finally, we introduced a novel loss function by incorporating a recently-introduced contextual loss [21] into the conventional L1 loss to enhance the image quality and correct for artifacts while minimizing the effect of potential large misalignments.

The images corrected by the network were evaluated using three commonly used image quality metrics: the mean absolute error (MAE), normalized root-mean-square deviation (NRMSE), and structural similarity index (SSIM). The MAE (ℓ1-norm) of pixel values has been frequently used to measure image quality for a long time. The MAE is based on the mean squared error (MSE; ℓ2-norm) and has been shown to outperform the MSE in image restoration tasks [22]. The NRMSE relates the RMSE to the observed range of the variable through normalization. The NRMSE is more sensitive to large errors than the MAE because the errors are squared before they are averaged. Although these MSE-based metrics are the simplest of all fidelity metrics, they have been criticized for their limited correlations with human perceptions of image quality [23]. The SSIM is a “perceptual” quality measure that considers image degradation as the perceived change in structural information, while also incorporating luminance masking and contrast masking phenomena [24]. Various aspects of the corrected images were also visually evaluated by expert clinicians.

We found that the proposed Contextual loss-Optimized Multi-Planar 2.5D U-Net (COMPUNet) significantly improved CBCT image quality, as validated by the three quantitative metrics described above. Clinical experts found that the resulting CBCT images showed fewer artifacts and less noise, without undue sacrifice of the sharpness and structural details in the original image, consistent with dental diagnostic purposes. Therefore, these novel procedures using deep neural networks may have applications in enhancing diagnostics in clinical practice.

## Material and methods

**Fig 1:**
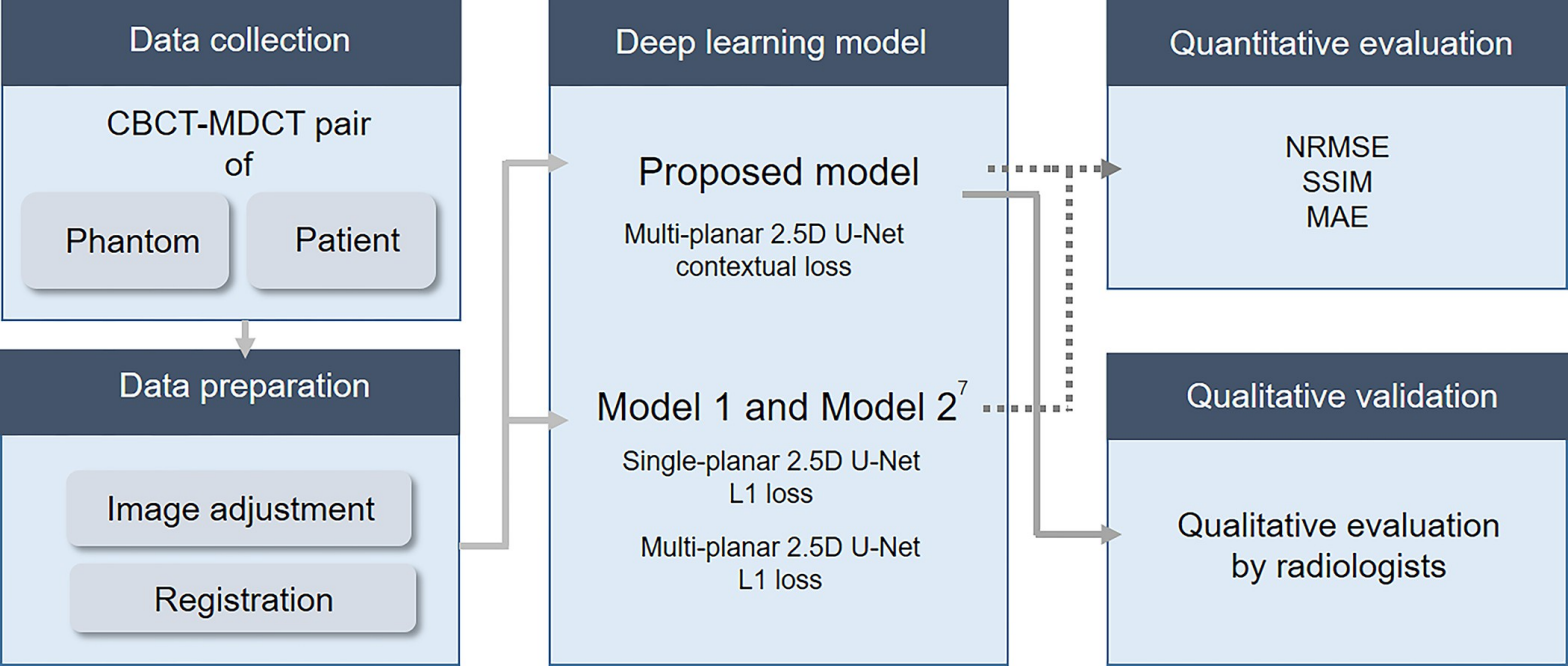
Schematic view of the overall work process.

We propose an approach to correct image artifacts in clinical CBCT images by using the corresponding MDCT scans as ground-truth high-quality images. Therefore, paired images of CBCT and MDCT from the same subjects were used in this study. The scanned images of anthropomorphic phantoms and the patient CBCT and MDCT data were both used for DL model development. This study was approved by the institutional review board (IRB) of Yonsei University Dental Hospital (IRB no. 2-2022-0024). The requirement for informed consent was waived due to the retrospective nature of this study, and all patient data were anonymized. The overall study workflow is described in Fig 1.

### Data collection

#### Clinical patient data

A clinical sample was retrospectively collected for training and testing the proposed model. In total, 30 CBCT-MDCT image pairs were retrieved from patients who underwent both CBCT and MDCT examinations at Yonsei University Dental Hospital between August 1, 2018 and August 31, 2021. MDCT and CBCT scans were taken for various purposes by different clinical departments, such as MDCT for sinusitis evaluation and CBCT for dental implant surgery. All image pairs were taken within a 2-month of interval, and image pairs with significant changes due to major surgery were excluded. The mean age of the subjects was 42.3 (± 19.0) years. Nine male and 21 female patients were included. All patients had more than one metallic restoration, including crowns, implant fixtures, inlays, bridges, and fixed retainers. No patients had an orthodontic bracket attached.

We used CBCT data obtained from two scanners manufactured by different vendors to improve the generalizability of the trained model. A set of 10 images was obtained using Rayscan Alpha Plus (Ray Co., Hwaseong, Korea) and a set of 20 images using Alphard 3030 (Asahi Roentgen Ind. Co. Ltd., Kyoto, Japan). All MDCT data were acquired with Optima CT520 (GE Healthcare, Chicago, IL, USA). The CBCT and MDCT images were taken with patients in different postures. The MDCT scans were taken with the patients lying down, whereas the CBCT scans were taken with the patients standing or sitting. Patients also often held a bite-block in the mouth during the CBCT examination. Therefore, the topology and relative position of the maxillary-mandibular and facial soft tissue were different between the two images, making image registration challenging (described in “Image Registration” in the Methods section). The scanner model and imaging parameters are described in Table 1. The clinical patient data were randomly split into training (40%; n = 12) and test (60%; n = 18) datasets.

**Table 1:** Exposure conditions for image data acquisition.

	Multi-detector computed tomography		
	Phantom data	Patient data	
Number of data sets	6	30	
Unit model	Sensation 64	Optima CT520	
Tube voltage	120 kVp	120 kVp	
Tube current	480 mAs	120 mAs	
Pitch	0.8	0.9	
Pixel size	0.37 mm	0.45 mm	
Slice thickness	0.60 mm	0.63 mm	
Display field of view	200 mm	200 mm	
CTDI_vol_	46.92 mGy	14.44 mGy	
	Cone-beam computed tomography		
	Phantom data	Patient data	
Number of data sets	6	10	20
Unit model	Rayscan Alpha Plus	Rayscan Alpha Plus	Alphard 3030
Tube voltage	80 kVp	80 kVp	80 kVp
Tube current	12 mA	12 mA	8 mA
Scan time	14 seconds	14 seconds	17 seconds
Voxel size	0.4 mm	0.4 mm	0.6 mm
Field of view	160 × 100 mm	160 × 100 mm	154 × 154 mm
DAP	1863 mGycm^2^	1863 mGycm^2^	2743 mGycm^2^

CTDI_vol_, computed tomography dose index; DAP, dose area product

#### Anthropomorphic phantom data

There were two obstacles in our method of correcting clinical CBCT images by using the corresponding MDCT as the ground truth high-quality image. First, it was difficult to perfectly register the two volumes due to differences in topology and the relative position of the maxillary-mandibular and facial soft tissue. Second, not every clinical MDCT image showed perfect image quality, often due to motion artifacts. We prospectively collected a phantom sample to alleviate these issues. Six pairs of CBCT-MDCT data were prepared with an anthropomorphic head phantom. CBCT images were acquired with Rayscan Alpha Plus (Ray Co. Ltd, Hwaseong, South Korea), and scanning was performed six times with different head positions by rotating 60° on the axial plane. For MDCT images, the radiation dose and pixel size were adjusted to obtain high-resolution images different from routine clinical images. The unit used was Sensation 64 (Siemens Medical Solutions, Forchheim, Germany). All phantom data were used as the training set.

#### Data preparation

We built an automated data preparation process involving intensive image registration and erroneous background masking, which aimed to minimize errors in image registration. As illustrated in Table 1, each CBCT and MDCT pair had a different resolution (pixel/voxel size and slice thickness), field of view (FOV), and orientation. Thus, an alignment (or registration) method is required to prepare the dataset. For this process, a 3D array of volume data was established from the DICOM images of CBCT and MDCT.

#### Image adjustment

The image orientation of MDCT was adjusted in terms of the right-anterior-superior orientation to match the CBCT scans. Next the MDCT image volumes were resampled to match the voxel size of the corresponding CBCT image data. For voxel intensity alignment, the background of CBCT was masked using the DIPY toolbox’s median *otsu* technique (Fig 1) [25]. The voxel intensities of CBCT were then matched with those of MDCT, converting into Hounsfield units (HUs). For both MDCT and CBCT, an intensity threshold of -1200 to 3071 HU was used, and data outside of this range were discarded.

#### Image registration

**Fig 2:**
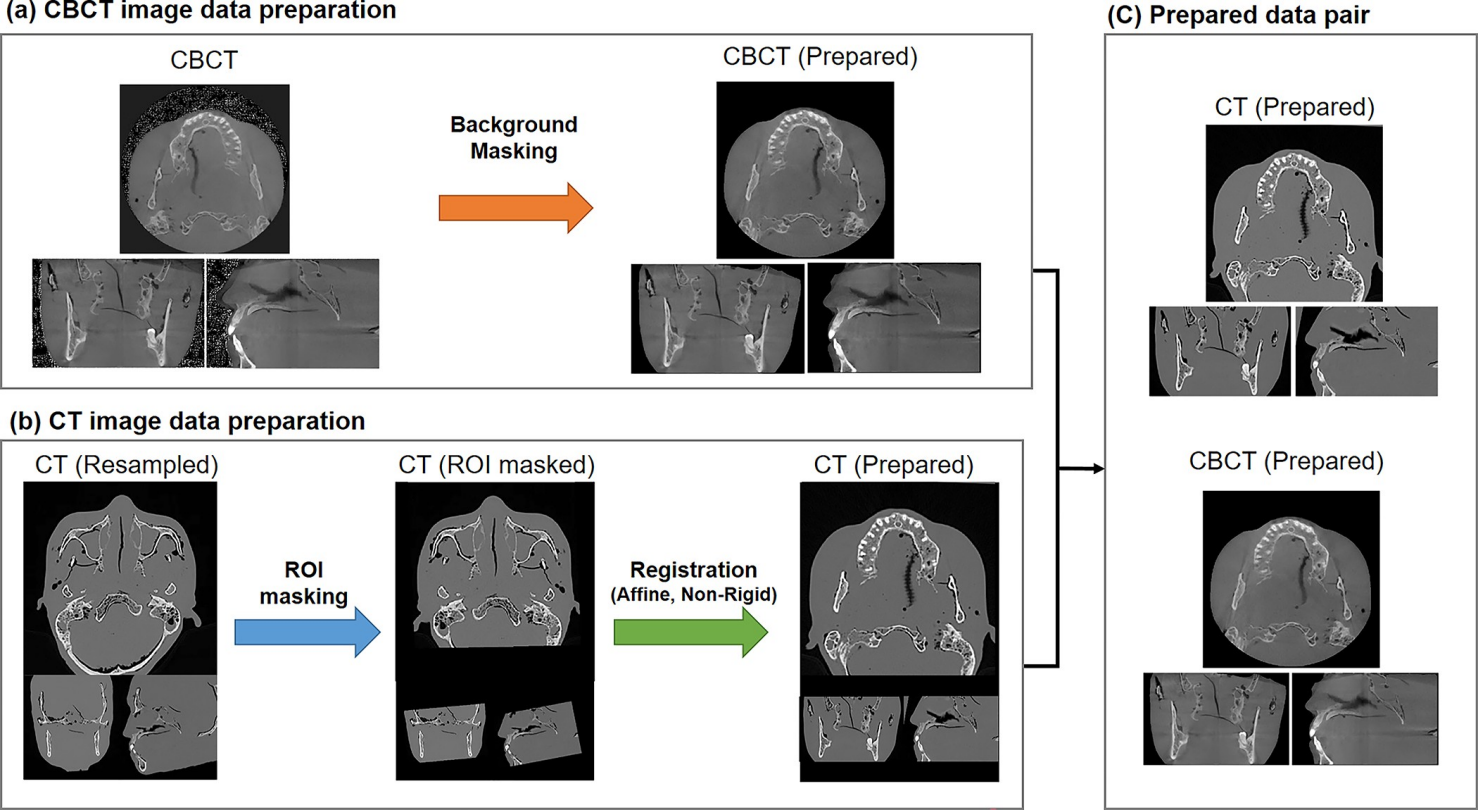
The data preparation process. CBCT images were generated by masking out the background signal noise (a). MDCT images were resampled to match the resolution of the CBCT images and then prepared using region-of-interest (ROI) masking and a two-step registration process utilizing affine and non-rigid registration to match the CBCT images (b). The final set of CBCT and MDCT image pairs was used for training and testing (c). CBCT, cone-beam computed tomography; MDCT, multidetector computed tomography.

Two techniques were used to register the MDCT volume image to the matching CBCT (Fig 2). Because the FOV of MDCT was larger than that of the CBCT data, the additional region was eliminated from the MDCT data for effective registration. In detail, the overlapping block (or ROI in Fig 2) between the MDCT and CBCT scans was obtained by the following process. 1) The CBCT volumetric image was registered to the MDCT scan through robust and fast rigid-transform-based registration. 2) The ROI of the MDCT volume was determined by identifying the overlapping block (cuboid) between the MDCT and CBCT scans. 3) Any ROI that did not correspond to this overlapping block was then masked, as demonstrated in Fig 2.

The alignment of MDCT to CBCT was then performed using affine registration (rotation, translation, and scaling) and fine-tuned using deformable registration. The Mattes mutual information metric [26] was used as the metric. Throughout this process, registration procedures from advanced normalization tools were used [27]. Finally, the CBCT and MDCT volume image pairings were prepared with the same orientation, resolution, and FOV.

### Deep learning model

**Fig 3:**
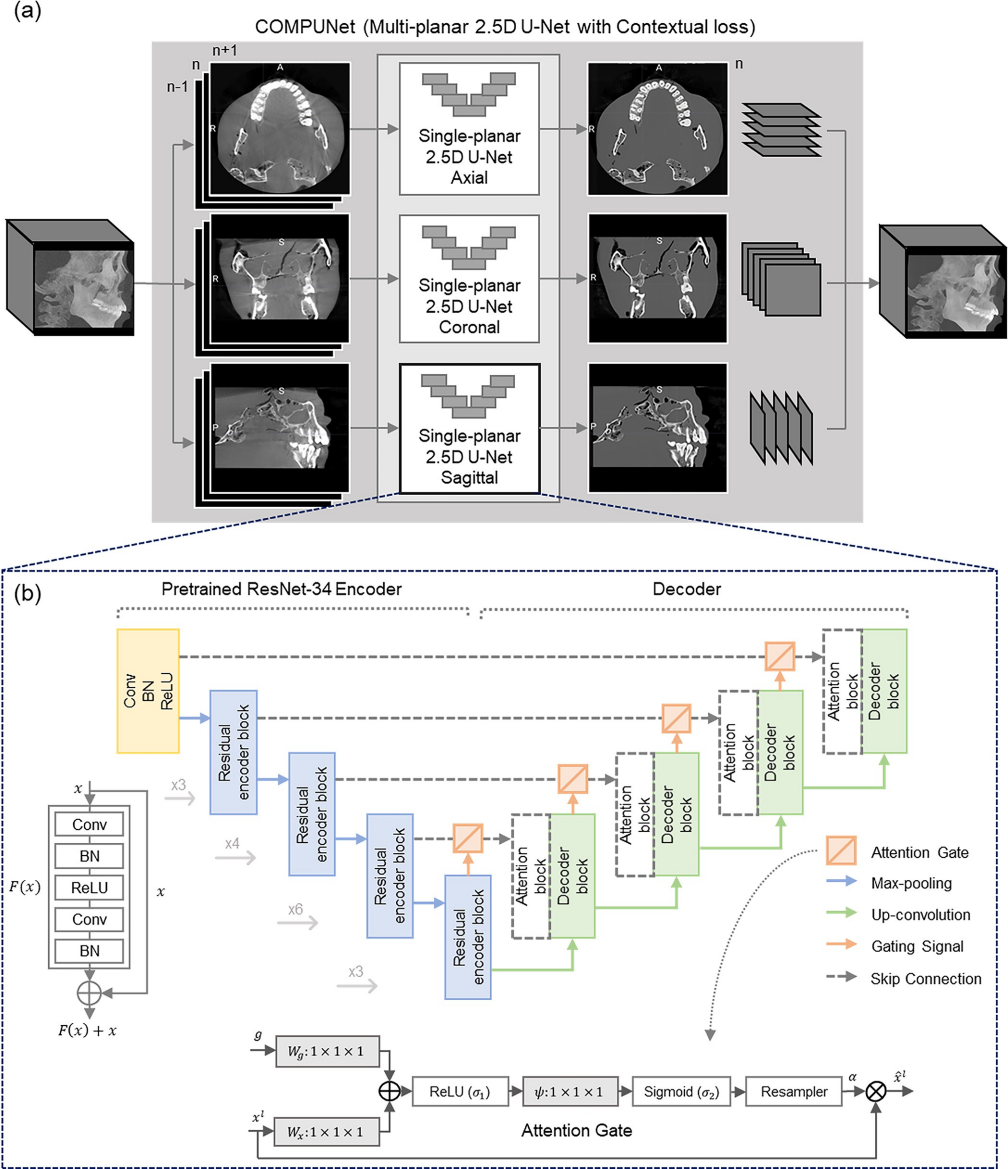
Schematic of COMPUNet (multi-planar 2.5D U-Net), which comprises three single-planar 2.5D U-Nets. (a) The architecture of a single-planar 2.5D U-Net with a ResNet-34 encoder and (b) attention modules. *x*^*l*^: input features, *α*: attention coefficients, *g*: gating signal collected from a coarser scale.

We developed a novel model, Contextual loss-Optimized Multi-Planar 2.5D U-Net (COMPUNet), by applying advanced techniques to resolve the issues of 2D and 3D U-Nets. We leveraged the recent 2.5D and multi-planar techniques to efficiently train the model with limited hardware resources while allowing a slight misalignment between the images. On top of that, a novel loss function was introduced by incorporating a recently-introduced contextual loss function along with conventional L1 loss to enhance the image quality and correct for artifacts while minimizing the effect of potential large misalignments. The network sliced each of the CBCT and MDCT volumes into multiple slices in three orthogonal directions: axial, coronal, and sagittal. The network was then trained using image slices in all three directions, referred to as multi-planar U-Net (Fig 3A). Each U-Net received three consecutive image slices (e.g., slices #N-1, #N, and #N+1) at once (reflecting the term “2.5D”) and generated a single corrected slice corresponding to the central image slice (e.g., slice #N). Then, during the inference phase, the outputs from the three different directions of the image were averaged to obtain a more robust estimate. The U-Net contained four encoder blocks and four decoder blocks (Fig 3B), and we used the pretrained ResNet34 [28, 29] (on ImageNet) as an encoder of each U-Net. In the decoder block, an attention gating method was employed for effective training [30]. For the network training, we introduced an additional loss function (contextual loss) to the conventional L1 loss function. Contextual loss is a recently introduced technique that uses the similarity between features rather than the pixel-wise distance function and is known to be effective for misaligned input-ground truth pairs [31]. This was thought to be advantageous for our study since misregistration errors between CBCT and MDCT images can result in blurring of the fine details of teeth, tooth-supporting structures, and trabecular bone.

#### Training strategy

The model proposed in this study was trained over 100 epochs with the Adam optimizer [32] and a learning rate of 0.001 to minimize the proposed loss (i.e., a combination of contextual and L1 loss). We randomly applied image augmentation to each image during neural network training to virtually increase the number of training examples. In each training iteration, we performed three types of augmentation.

- Random flips on the horizontal and vertical axes.
- Random rotations between -90° and 90°.
- Cropping at a random location to 240×240 size.

PyTorch 1.8.0 [33] was used for implementation and training. The training dataset included 18 pairs of CBCT–MDCT data, including 6 phantoms and 12 patients. To compare the model performance, two additional models (Model 1 and Model 2) were trained. Those models were introduced in previous studies [7], and the model in this study can be described as follows:

- Model 1: A single-planar 2.5D U-Net with L1 loss
- Model 2: A multi-planar 2.5D U-Net with L1 loss
- COMPUNet: A multi-planar 2.5D U-Net with L1 and contextual losses

#### Performance evaluation

*Quantitative evaluation*. The image quality of each network’s output was evaluated using quantitative image assessment indices. The assessment was conducted on 18 test data sets using three image quality metrics: NRMSE, SSIM, and MAE. For each volume in the test-set *y* = {*y*_1_, *y*_2_,…,*y*_*N*_} and $\hat{y}=\{{\hat{y}}_{1},{\hat{y}}_{2},\ldots ,{\hat{y}}_{N}\}$, the three metrics are defined as follows:

$$NRMSE=\frac{\sqrt{\sum_{i=1}^{N}{\left({\hat{y}}_{i}-y_{i,k}\right)}^{2}}}{{\hat{y}}_{i}^{max}-{\hat{y}}_{i}^{min}}$$ (1)

$$SSIM=\frac{\left(2\mu_{{\hat{y}}_{i}}\mu_{y_{i,k}}+c_{1})(2\sigma_{{\hat{y}}_{i}y_{i,k}}+c_{2}\right)}{\left(\mu_{{\hat{y}}_{i}}^{2}+\mu_{y_{i,k}}^{2}+c_{1})(\sigma_{{\hat{y}}_{i}}^{2}+\sigma_{y_{i,k}}^{2}+c_{2}\right)}$$ (2)

$$MAE=\frac{1}{N}\sum_{i=1}^{N}\left|{\hat{y}}_{i}-y_{i,k}\right|$$ (3)

where *y* and $\hat{y}$ are MDCT and DL outputs, respectively. *N* denotes the number of paired data and *k* denotes the DL models used. *μ*_*y*_ and $\mu_{\hat{y}}$ are the averages of *y* and $\hat{y}$, respectively, $\sigma_{y}^{2}$ and $\sigma_{\hat{y}}^{2}$ are the variances of *y* and $\hat{y}$, respectively, and $\sigma_{\hat{y}y}$ is the covariance of *y* and $\hat{y}$. *c*_1_ = (*k*_1_*L*)^2^ and *c*_2_ = (*k*_2_*L*)^2^ are used to stabilize division with a weak denominator, where L is the dynamic range of the pixel values and *k*_1_ = 0.01 and *k*_2_ = 0.03 are used. A smaller NRMSE value, a smaller MAE value, and a larger SSIM value indicate superior image quality.

*Qualitative validation*. For the 18 original CBCT (oCBCT) and corresponding predicted (or corrected) CBCT (pCBCT) images, the evaluation was conducted using a PACS viewer (Zetta, Tae-young, South Korea) in random order. Two radiologists made an evaluation using a modified version of the clinical CBCT image evaluation chart provided by the Korean Academy of Oral and Maxillofacial Radiology (Table 2) [13]. The evaluation procedure was conducted individually in blind condition. The clinical CBCT image evaluation chart was composed of three criteria (artifacts, noise, and contrast) of 10 evaluation items. Individual items could be scored as 1 = poor quality, 2 = moderate, and 3 = good quality. The artifact criterion was assessed using 3 items with a perfect score of 9 points. The noise criterion was evaluated using 2 items and the contrast criterion was assessed using 5 items. Each item received a score between 0 and 3 and the maximum total score was 30 points. The overall image grade was also assessed as 0 = image quality with no diagnostic value, 1 = poor but feasible to diagnose, 2 = moderate, and 3 = good image.

**Table 2:** Clinical CBCT image evaluation chart.

	Severe (1)	Moderate (2)	Few (3)
[Artifacts] 1. Streak artifact 1–1. Mandibular ramus area 1–2. Zygomatic process area 2. Ring artifact 3. Blurring edge and shading 3–1. Mandibular angle area			
[Noise] 4. Noise on mouth floor 5. Noise on maxillary sinus			
	Unclear (1)	Moderately defined (2)	Clear (3)
[Resolution] 6. Enamel, dentin, pulp (upper molar and lower molar) 7. PDL space and lamina dura (upper molar and lower molar) 8. Sinus floor 9. Bone pattern 9–1. Mastoid air cell bone structure 9–2. Trabecular bone of mandible 10. TMJ complex			
[Overall grade of image]			
No diagnostic value (0) Poor (1) Moderate(2) Good (3)			

CBCT, cone-beam computed tomography; PDL, periodontal ligament; TMJ, temporomandibular joint.

### Statistical analysis

For a quantitative evaluation of the model proposed in this study, Model 1, and Model 2, the mean values of NMRSE, SSIM, and MAE were compared among the images generated with the three models and the oCBCT using analysis of variance with a 95% confidence interval. When there was statistical significance, the metrics of the oCBCT were compared to those of all three models using the *t*-test while correcting for multiple comparisons. For the qualitative evaluation, the interobserver reliability was assessed using the Cohen’s kappa coefficient (κ). Scores were compared between the oCBCT and pCBCT using the Wilcoxon signed-rank test according to the artifacts, noise, and resolution criteria.

## Results

**Fig 4:**
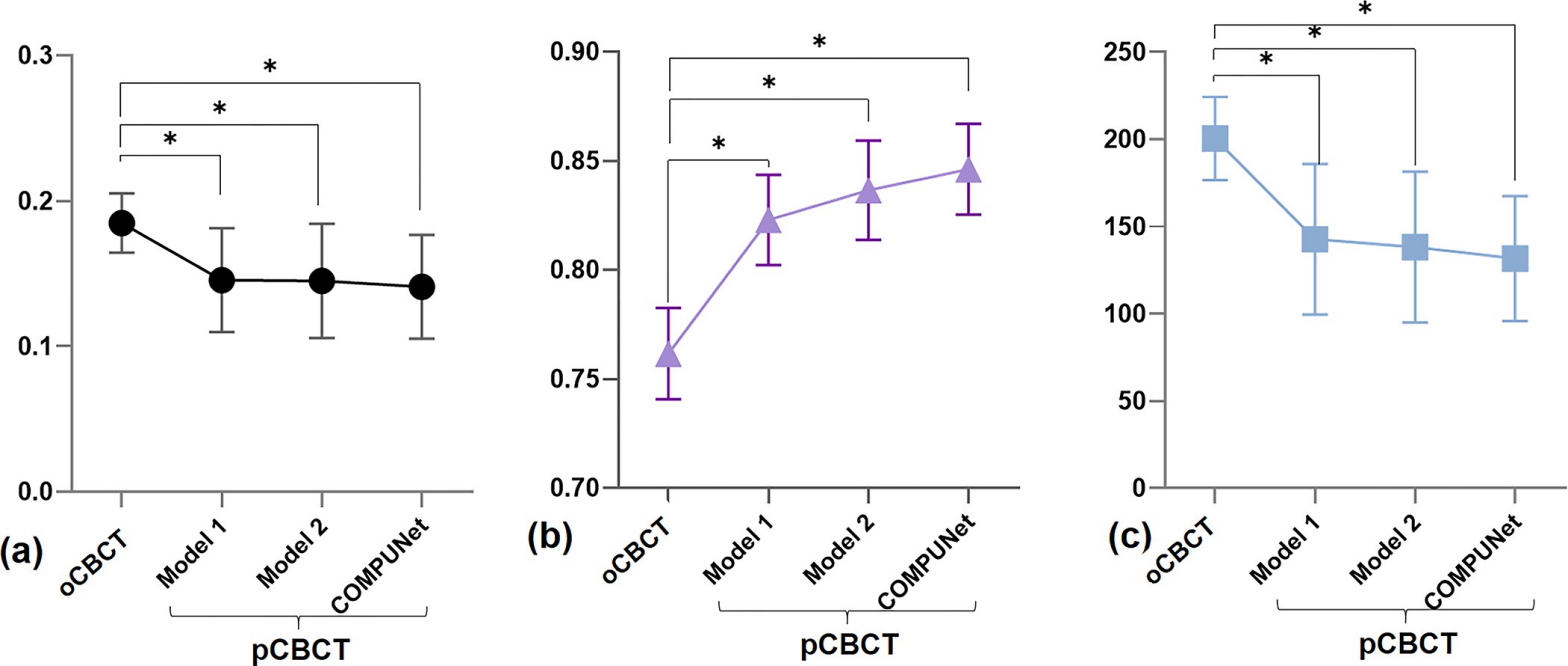
Quantitative evaluation through an ablation study. NRMSE (a), SSIM (b), and MAE (c) for comparisons between the original (oCBCT) and predicted CBCT (pCBCT), showing that the best-performing model was the proposed model compared to Model 1 and Model 2. (* p < 0.005).

**Fig 5:**
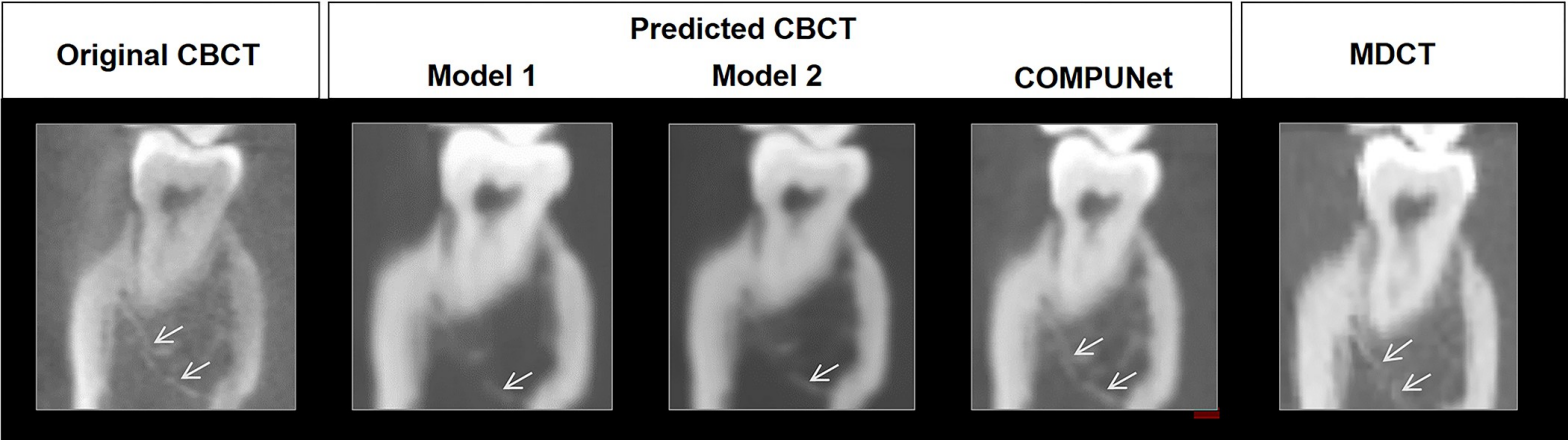
The trabecular bone pattern (arrow) in the same image slides. The fine bone details are best preserved in the predicted CBCT of the proposed model, COMPUNet, compared to that of Model 1 and Model 2.

The NRMSE, SSIM, and MAE values of the pCBCT of the proposed model, COMPUNet, were all significantly improved compared with the oCBCT. The pCBCT of the proposed model also showed significantly better performance than that of Model 1 and Model 2, in terms of NRMSE, SSIM, and MAE values (Fig 4). Fig 5 shows that the fine bone details were only preserved in the pCBCT of the COMPUNet, unlike the other models.

**Fig 6:**
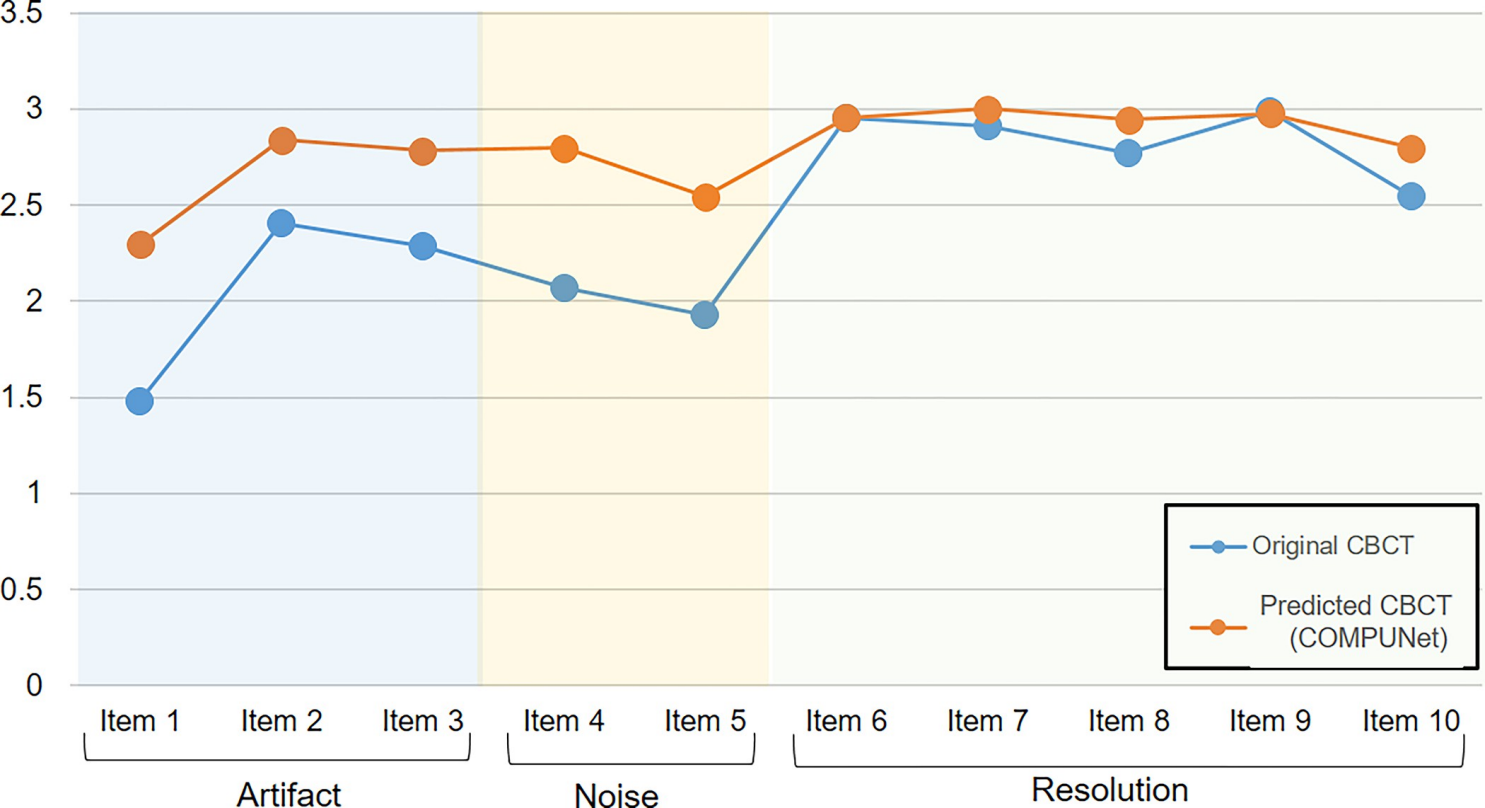
Mean scores of individual items in the clinical CBCT evaluation chart. Items for the artifacts and noise criteria showed improvements in the pCBCT images, while those for the resolution criterion improved less.

**Fig 7:**
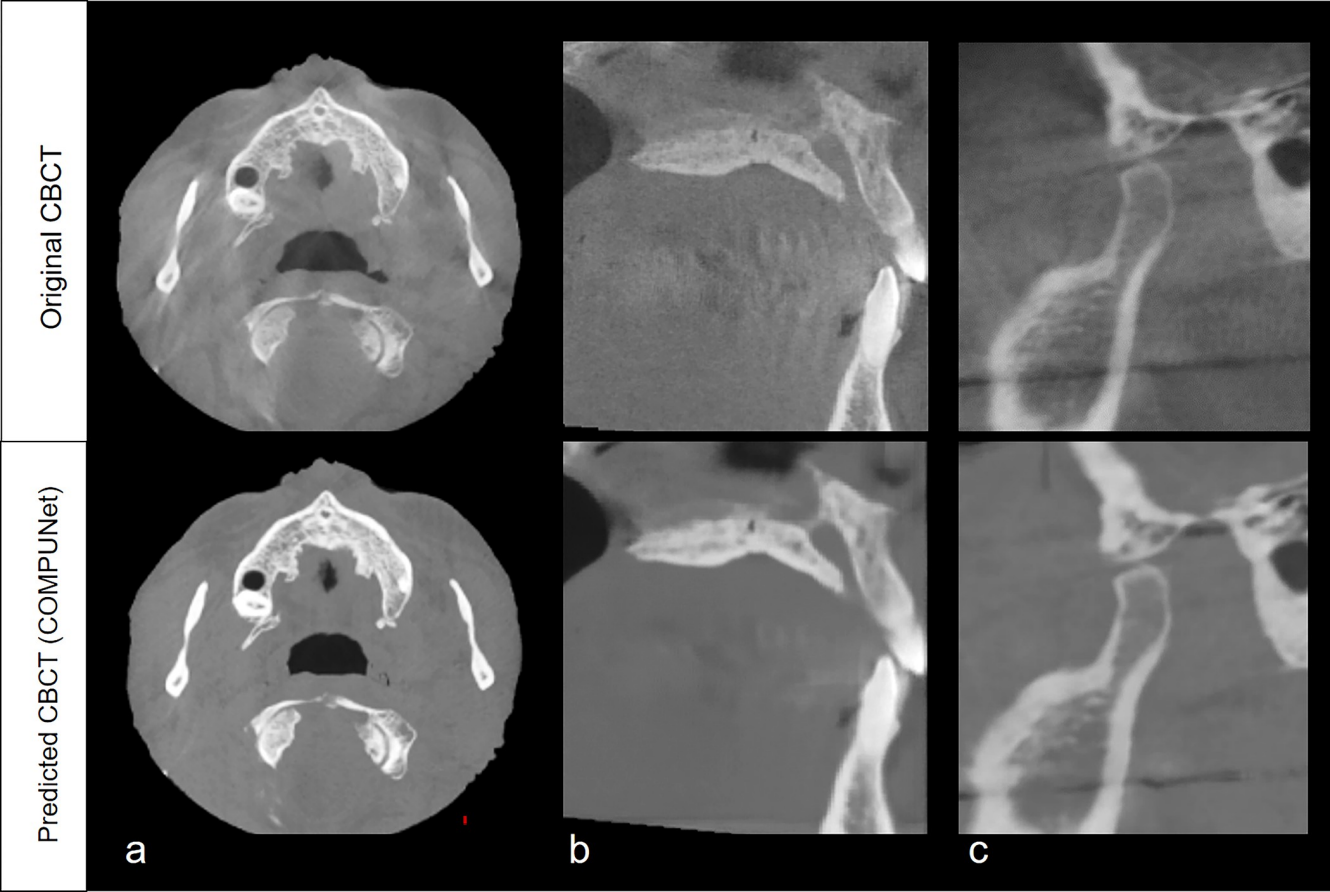
Original CBCT with an overall image grade of poor and the corresponding predicted CBCT image. (a) The maxilla in an axial view (b), the anterior teeth region in a sagittal view, and (c) the temporomandibular region in a parasagittal view show reduced artifacts and noise in the COMPUNet CBCT compared to the original CBCT images.

**Fig 8:**
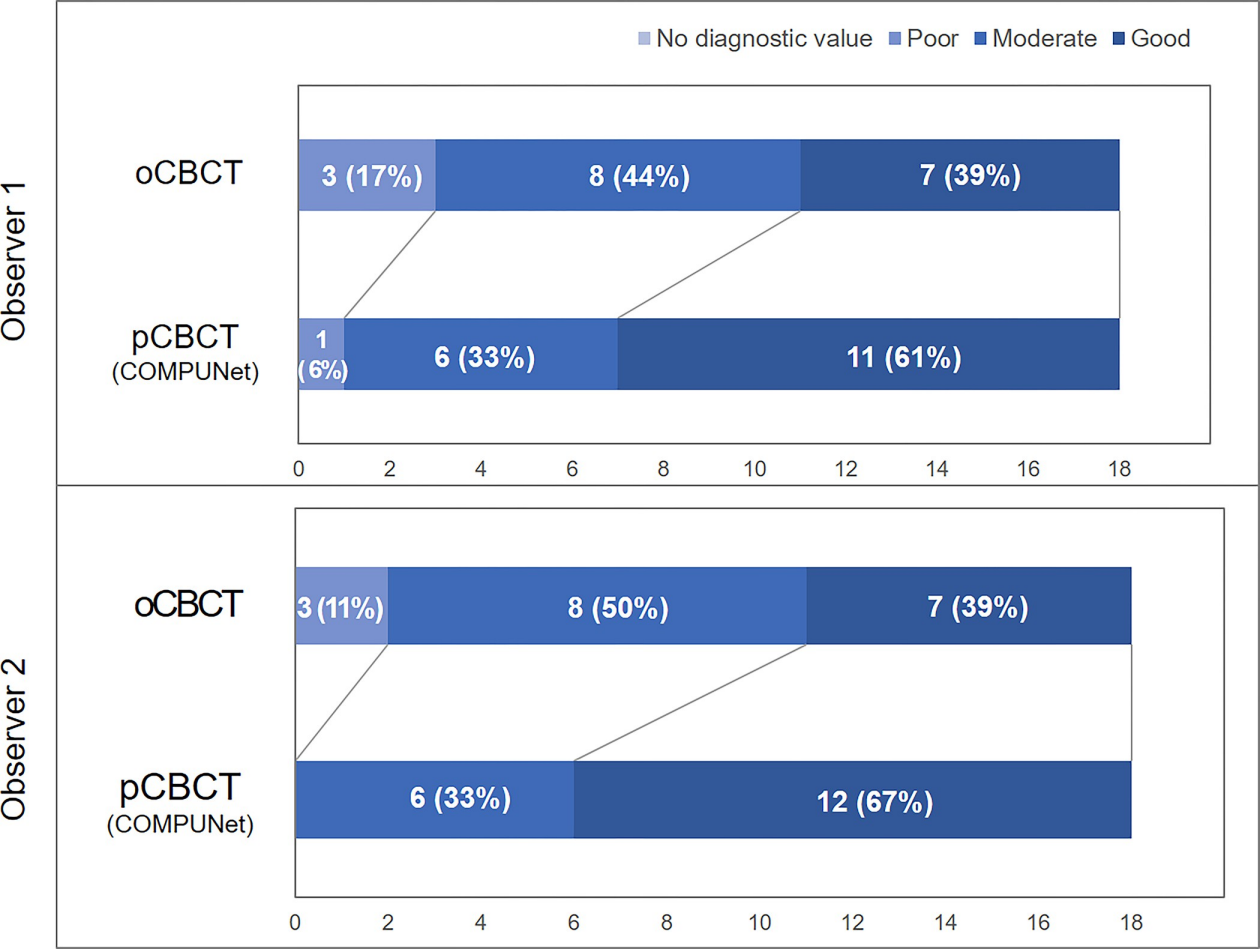
Distribution of overall image quality evaluation grades. The proportion of CBCT with poor grades decreases (sky blue) and those with good grades increases (dark blue) in the predicted CBCT (pCBCT) compared to the original CBCT (oCBCT) for both observers.

The interobserver reliability for the qualitative evaluation showed good to excellent agreement (κ = 0.87, *P*<0.05). For the artifacts, noise, and contrast criteria, the scores were significantly different between the oCBCT and pCBCT of COMPUNet. The total score (i.e., the sum of items for each criterion) was also significantly higher for pCBCT than for the oCBCT (Table 3). For the individual evaluation items, most items showed higher scores in the pCBCT, except for item 6 (enamel, dentin, pulp) and 9 (bone pattern) in the contrast section, which showed almost the same score (Fig 6). There were more images with good grades in the pCBCT of COMPUNet than in the oCBCT. Moderate and poor grades were presented more frequently for the oCBCT images than for the pCBCT images (Figs 7 and 8).

**Table 3:** Scores from the qualitative evaluation.

	Original CBCT	Predicted CBCT	*P*-value
Artifacts (9 points)	6.14 ± 0.79	7.91 ± 0.79	0.000*
Noise (6 points)	3.95 ± 0.56	5.28 ± 0.41	0.000*
Resolution (15 points)	14.09 ± 1.19	14.64 ± 0.77	0.001*
Total (30 points)	24.18 ± 1.91	27.83 ± 1.55	0.000*

* Wilcoxon signed-rank test, *p*-value < 0.05.

## Discussion

With the growth of digital dentistry and the use of 3D images, the scope of CBCT application has grown significantly during the last decade. However, due to pronounced artifacts, its use has been limited, impairing the diagnostic utility and precision of 3D models for surgical simulation [34]. Although numerous deep learning methods have tackled the challenge of removing artifacts in CBCT, their usage in dentistry has posed several challenges due to the difficulty of preparing training datasets (i.e., aligning MDCT and CBCT data acquired from diverse scanning conditions), and the loss of fine details due to blurring. The present study is the first to attempt to solve these issues using a multi-step registration process and novel network architecture with a multi-planar 2.5D U-Net-based network and a carefully designed loss function.

By employing the multi-planar method, it was possible to eliminate artifacts that were difficult to correct with a single-planar network. The present study used three 2.5D single-planar U-Nets, trained in the axial, coronal, and sagittal directions, respectively, and averaged across the three generated volumes. This was advantageous for minimizing streaking artifacts, which can be difficult to distinguish in one orientation but are readily discernible in the other two. Some artifacts were more visible in certain orientations than the others (often depending on the anatomical structure present in the orientation). By complementing one another, the multi-planar network removed artifacts more efficiently than the single-planar network. As a result, the NRMSE decreased from 0.1455 to 0.1450, the SSIM increased from 0.8228 to 0.8365, and the MAE decreased from 142.6 to 138.1.

By incorporating a contextual loss term, which is computed on a per-instance basis on the feature map error, it was possible to recover blurred fine features compared with the conventional L1 loss alone. Our loss function was advantageous in the presence of misalignment as it made no assumptions that the input image and the target image were perfectly aligned. It extracted contextual features from each image using a pretrained VGG19 network, located corresponding features, and analyzed their similarity. As a result, the SSIM increased (from 0.8365 to 0.8461) and the error values decreased (NRMSE from 0.1450 to 0.1410, MAE from 138.1 to 131.6). As seen in Fig 6, the CBCT images’ artifacts and noise were reduced, but the intricate and fine structural details were maintained. Additionally, experts confirmed that the suggested method preserved fine details well, as the ratings for fine details (referred to as resolution) were higher in the pCBCT than in the oCBCT.

The clinical evaluation demonstrated that the proposed CBCT enhancement improved the visibility of the sinus floor and the TMJ complex, which are critical structures for diagnosis. Since the majority of the upper teeth protrude into the maxillary sinus, identifying the maxillary floor on CBCT prior to tooth extraction is critical for avoiding sinus surgery [35]. Additionally, the distance between the tooth root and the sinus floor impacts the effectiveness of dental implant surgery [36], and dental clinicians have expressed interest in being able to clearly define the sinus floor on CBCT. For the examination of TMJ disease, CBCT is preferred over MDCT as a general strategy for the current clinical situation [37]. Thus, image enhancement, particularly of certain anatomic regions in our investigation, has significant clinical implications.

While the current work demonstrates an intriguing technical development in deep learning for improving CBCT images, the study’s scope is limited. We have demonstrated that the proposed technique preserves fine details on CBCT, but does not improve them. More specifically, the increase in the score for resolution was less than the increase in the scores for artifacts and noise. This is mostly due to the fact that the tooth structures (enamel, dentin, and pulp) and bone patterns (mastoid air cell and trabecular bone) stayed constant rather than improving. Although the model effectively reduced various artifacts, including beam hardening artifacts and metal artifacts, they were not completely removed—especially when the original quality was quite poor (e.g., Fig 8) or when the artifact patterns were not common in the training dataset. It should be kept in mind that we designed the loss function of the model so that the texture and fine details in CBCT would not vanish. We observed that artifacts could be further removed by adjusting weights to each term in the loss function; however, this resulted in removing the CBCT texture and making it look artificial around the soft tissue in many cases. Therefore, the model can be easily tuned to remove artifacts more aggressively as needed.

More thorough dataset preparation may make it possible to improve the fine details. Due to the study’s retrospective design, the clinical CBCT and MDCT images were taken with different patient postures. Specifically, some patients bit on the bite-block while standing or sitting during the CBCT examination. In contrast, the MDCT scans were taken with the patient lying down. As standing or sitting alters the relative position and topology of the maxillary-mandibular and facial soft tissue shapes, perfect registration between the pairs of images was difficult. More rigorous dataset preparation, such as realistic phantom scanning or dedicated image acquisition design, may be advantageous for further improving fine details. We will also continue to collect more CBCT-MDCT pairs to obtain a larger training dataset. This research direction might have clinical value for analyzing bone morphology and trabecular structure, which are critical for dental surgical procedures in order to determine the ideal placement of dental implants and monitor the bone healing process. Through the examination of bone patterns, CBCT images have proven their efficacy in predicting the prognosis of dental surgery [38, 39].

## Conclusion

In conclusion, we proposed a novel technique for improving the quality of CBCT images for dental diagnostic and treatment planning purposes. The significant improvement in CBCT image quality was validated by comparing the derived CBCT images to the original CBCT images using NMRSE, SSIM, and MAE values. Clinical experts additionally evaluated the resulting CBCT images as having fewer artifacts and less noise, while keeping the high resolution and sharpness of the original image. The DL method validated in the current study is suggested as a tool with practical applicability in actual clinical practice.
